# The time resolution of the St Petersburg paradox

**DOI:** 10.1098/rsta.2011.0065

**Published:** 2011-12-13

**Authors:** Ole Peters

**Affiliations:** 1 Department of Mathematics and Grantham Institute for Climate Change, Imperial College London, London SW7 2AZ, UK; 2 Department of Atmospheric and Oceanic Sciences, University of California, Los Angeles, 7127 Math Science Building, 405 Hilgard Avenue, Los Angeles, CA 90095-1565, USA; 3 Santa Fe Institute, 1399 Hyde Park Road, Santa Fe, NM 87501, USA

**Keywords:** St Petersburg paradox, expectation, ergodicity, risk, utility

## Abstract

A resolution of the St Petersburg paradox is presented. In contrast to the standard resolution, utility is not required. Instead, the time-average performance of the lottery is computed. The final result can be phrased mathematically identically to Daniel Bernoulli's resolution, which uses logarithmic utility, but is derived using a conceptually different argument. The advantage of the time resolution is the elimination of arbitrary utility functions.

## 1. Introduction

I investigate practical consequences of a radical idea built into the foundations of probability theory. The idea is that of embedding a stochastic system in an ensemble of systems, which all start in the same state, but develop along different trajectories. To understand how this idea was absorbed into the theory, the original motivation for developing the concept of probability and expectation values is reviewed in §2. Section 3 describes the St Petersburg paradox, the first well-documented example of a situation where the use of ensembles leads to absurd conclusions. Daniel Bernoulli's [1] response to the paradox is presented in §4, followed by a reminder of the more recent concept of ergodicity in §5, which leads to an alternative resolution in §6 with the key theorem 6.2. Section 7 explains the intriguing relation of this mathematically similar but conceptually wholly different resolution to Bernoulli's work and resolves difficulties with unbounded utility functions noted by Menger [2]. Section 8 concludes that the prominence in economics of Bernoulli's early arguments may have contributed to poor risk assessments of modern financial products, with consequences for market stability through the effect of credit and leverage, as foreseen by writers as early as Adam Smith.

## 2. Origins of probability theory

Formal and concrete concepts of likelihood were first developed in the context of gambling—notable are the works by Pacioli [3], by Cardano in the mid-sixteenth century [4], and the work by Pascal and Fermat in the summer of 1654. A prominent question treated by Pacioli [3] as well as by Pascal and Fermat (B. Pascal & P. Fermat 1654, personal communication between themselves) is the following ‘problem of the points':^1^ imagine that a game of dice has to be abandoned before it can be concluded. For instance, players may be betting money on the highest score in rolling a dice three times, but have to leave after two throws. In this situation, how is the ‘pot’, the total wager, to be distributed among the players in a fair manner?

The first observation is that this is a moral question. Mathematics may aid in answering it, but cannot resolve it without appealing to external information, as any answer must depend on the concept of fairness. It could be perceived as fair that the player with the most points is entitled to the total wager. Another concept of fairness would be to call the game inconclusive and return to each player his or her individual wager, or the pot may be split equally between the participants. Apparently, at least until the seventeenth century, there was no universal agreement on the relevant concept of fairness. Pacioli [3], for instance, argued that the fair solution is to divide the pot in proportion to the points that each player has accrued when the game is interrupted [see [5, p. 15].

A century and a half later, Pascal was approached by Chevalier de Méré to produce a conclusive argument based on mathematics that would settle the issue. Pascal and Fermat corresponded on the subject and agreed that the fair solution is to give to each player the expectation value of his winnings. The expectation value they computed is an ensemble average, where all possible outcomes of the game are enumerated, and the products of winnings and probabilities associated with each outcome for each player are added up. This procedure uses the then revolutionary idea of parallel universes. Instead of considering only the state of the universe as it is, or will be, an infinity of additional equally probable universes is imagined. Any of these additional universes, for all we know, could be reality (i.e. the world as it will be). The proportion of those universes where some event occurs is the probability of that event. We will see that in the eighteenth century Bernoulli noticed undesired properties of the ensemble average, and in the nineteenth century Boltzmann began to specify conditions for its applicability.

The 1654 investigation, which is generally considered the beginning of probability theory, was concerned with a specific problem. It did not attempt to make any predictions, for instance involving repetitions of the game, but solely gave quantitative guidelines where individuals had incompatible moral intuitions. Moral considerations were certainly at the heart of the early debate. Pascal famously used expectation values to argue in favour of his religious beliefs, and much of Cardano's work on gambling is concerned with morals. He came very close to defining a fair game as one where no player has an expected advantage over others: ‘To the extent to which you depart from that equality, if it is in your opponent's favour, you are a fool, if in your own, you are unjust’ [4, p. 189].

Following Pascal's and Fermat's work, however, it did not take long for others to recognize the potential of their investigation for making predictions. Halley [6], writing in these pages 318 years ago, built on earlier work by Graunt [7], and devised a method for pricing life annuities. The idea of embedding reality in infinitely many possible alternatives was revolutionary in 1654, it was essential in the development of statistical mechanics in the nineteenth century [8,9], and it continues to be a fruitful means of conceptualizing complex and stochastic systems [10]. Nonetheless, the idea itself is a dubious philosophical construct, justified empirically by the success that, under appropriate conditions, comes with allowing the use of mathematical rigour. Historically, it seems that the philosophical weakness was initially ignored in applications. In §5, we will review an alternative conceptualization of randomness.

Huygens [11] is credited with making the concept of expectation values explicit and with first proposing an axiomatic form of probability theory. This was helpful in developing the field mathematically, as results could now be proved to be correct. On the other hand, by introducing an axiomatic system, correctness becomes restricted to the context of the axioms themselves. A proven result in probability theory follows from the axioms of probability theory, now usually those of Kolmogorov [12]. It is related to reality only insofar as the relevant real conditions are reflected by the axioms. Kolmogorov [12, pp. 1–2] wrote ‘The theory of probability […] should be developed from axioms in exactly the same way as Geometry and Algebra. This means that after we have defined the elements to be studied and their basic relations, and have stated the axioms by which these relations are to be governed, all further exposition must be based exclusively on these axioms, independent of the usual concrete meaning of these elements and their relations'. He wrote that it would be a different ‘aim […] to tie up as closely as possible the mathematical theory with the empirical development of the theory of probability’.

To summarize: the first systematic investigation into stochastic systems was concerned with moral advice. The second established an axiomatic system.

## 3. The lottery

The St Petersburg paradox was first put forward by Nicolaus Bernoulli in 1713 [13, p. 402]. He considered lotteries of the following type:

A fair coin is tossed.

(1) On heads, the lottery pays $1, and the game ends. On tails, the coin is tossed again.
(2) On heads, the lottery pays $2, and the game ends. On tails, the coin is tossed again.
 ⋮
(*n*) On heads, the lottery pays $2^*n*−1^, and the game ends. On tails, the coin is tossed again.
 ⋮

In other words, the random number of coin tosses, *n*, follows a geometric distribution with parameter 1/2, and the payouts increase exponentially with *n*. We may call *n* a ‘waiting time’, although in this study it is assumed that the lottery is performed instantaneously, i.e. a geometric random variable is drawn and no significant physical time elapses. The expected payout from this game is
3.1
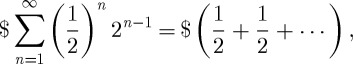
which is a diverging sum. A rational person, N. Bernoulli argued, should therefore be willing to pay any price for a ticket in this lottery. In reality, however, people are rarely willing to pay more than $10, which constitutes the paradox.

Reactions to the paradox include the following.

Even though the expected payout is infinite, there is not an infinite amount of money or goods in the world to pay up. So the lottery is not realistic (G. Cramer 1728, personal communication with N. Bernoulli). If the payouts are limited to some realistic value, then the lottery's expected payout is drastically reduced. For example, the 31st term in the sum, equation (3.1), comes from a payout of about $10^9^, so limiting payouts to $10^9^ reduces the expected payout from $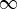 to $15. Similarly, one could argue that it is only too sensible to ignore events with a probability of the order of 10^−9^ [2].

Another argument is that no one would offer such a lottery because it carries an infinite expected loss for the lottery seller, which makes it irrelevant [14].

## 4. Bernoulli's resolution

The quantity calculated in equation (3.1) is usually called an ‘expected’ payout. But as it fails to capture the reality of the situation its conceptual validity must be questioned. Bernoulli [1, p. 1] noted

Paragraph 1. Ever since mathematicians first began to study the measurement of risk there has been general agreement on the following proposition: expected values are computed by multiplying each possible gain by the number of possible cases where, in this theory, the consideration of cases which are all of the same probability is insisted upon.

Indeed, Huygens [11] had postulated: ‘if any one should put 3 shillings in one hand without telling me which, and 7 in the other, and give me choice of either of them; I say, it is the same thing as if he should give me 5 shillings…’. This concept of expectation is agnostic regarding fluctuations, which is harmless only if the consequences of the fluctuations, such as associated risks, are negligible. This is usually the case in small-stakes recreational gambling as considered in the earliest studies of chance by Pacioli [3], Cardano [4] and Fermat and Pascal (P. Fermat & B. Pascal 1654, personal communication between themselves), mentioned in §2, but it is not the case in the St Petersburg paradox. Noticing that the ability to bear risk depends not only on the risk but also on the riskbearer's resources, Bernoulli [1, p. 24] wrote under paragraph 3:

If I am not wrong then it seems clear that all men cannot use the same rule to evaluate the gamble. The rule established in paragraph 1 must, therefore, be discarded.

Bernoulli, and shortly before him Cramer (G. Cramer 1728, personal communication with N. Bernoulli), drew attention to psychological and behavioural issues involved in the evaluation of the proposed lottery. The desirability or ‘utility’ associated with a financial gain, they argued, depends not only on the gain itself but also on the wealth of the person who is making this gain. Instead of computing the expectation value of the monetary winnings, they proposed to compute the expectation value of the gain in utility. To this end, the utility function *u*(*w*) was introduced, which specifies the utility of a wealth of $*w*.

As an extra dollar is generally worth less to a rich person than to a poor person, *u*(*w*) is assumed to be concave, such that d*u*(*w*)/d*w* is monotonically decreasing. While exceptional circumstances can render this assumption invalid (Bernoulli cites an imprisoned rich man who only needs another 2000 ducats to buy his freedom), it is well confirmed behaviourally. Otherwise, *u*(*w*) is only loosely constrained. Bernoulli [1] suggested the logarithmic function, 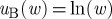, whereas Cramer (G. Cramer 1728, personal communication with N. Bernoulli) had proposed using 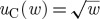 instead. Bernoulli's proposition of the logarithm was based on the intuition that the increase in wealth should correspond to an increase in utility that is inversely proportional to the wealth a person already has, d*u*/d*x*=1/*x*, whose solution is the logarithm.

Bernoulli [1] thus ‘discarded the rule’ (for calculating expected gains in wealth) by replacing the object whose expectation value was to be calculated. Instead of gains in wealth, he decided to focus on the expectation of gains in some function of wealth.

In §6, we will also discard the rule established in paragraph 1 of Bernoulli [1], but not by replacing the object whose average is to be calculated, i.e. not by replacing plain monetary gains by a function of those gains. We will replace the type of average, using the time average instead of the ensemble average. This is necessary because the system under investigation (the dynamics of monetary wealth) is not ergodic, as will be shown in §6. In doing so, we will critique the implicit considering of multiple imagined systems, or parallel universes.

But first, applying Bernoulli's reasoning, we compute the expected change in logarithmic utility, 〈Δ*u*_B_〉, owing to playing the lottery, given the initial wealth $*w* and the cost of a ticket in the lottery $*c*,
4.1
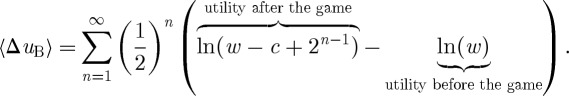
This sum converges (as long as each individual term is finite) as is readily shown using the ratio test. Depending on *w* and *c*, the quantity can be positive or negative, reflecting expected gain or loss of utility. Assuming that potential lottery players base their decisions not only on the expected monetary gain, but instead also on the expected gain in usefulness, and that usefulness is appropriately represented by *u*_B_, the paradox is thus resolved.

It is dissatisfying that this resolution of the paradox relies on a function *u*(*w*) that is postulated and, in the framework of Cramer and Bernoulli, cannot be derived from more fundamental considerations. Disagreements on whether the assumptions (the characteristics of diminishing marginal utility of wealth) are realistic are difficult to settle. Anticipating this objection, Bernoulli [1, p. 31]—Daniel being perhaps less mathematician than scientist—appealed to observations: ‘Since all our propositions harmonize perfectly with experience it would be wrong to neglect them as abstractions resting upon precarious hypotheses'.

The responses to the paradox mentioned at the end of §3 are similarly dissatisfying—they address the relevance of the problem and argue that it would never really arise, but they do not resolve it. As the paradoxical aspect is the behaviour of real people, however, these arguments are valid, and all means of disposing of the paradox could be similar in character.

While Bernoulli's observations of human risk aversion and even the functional form he proposed for modelling these are ‘correct’ in a specific sense elaborated in §6, these behavioural regularities have a physical reason that Bernoulli failed to point out. In fact, it appears that he was not aware of this physical reason, which justifies only 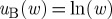. Bernoulli [1, p. 33] did not consider the logarithmic form of utility essential and wrote of Cramer's work, which uses 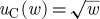: ‘Indeed I have found his theory so similar to mine that it seems miraculous that we independently reached such close agreement on this sort of subject’.

## 5. Ergodicity

The question of ergodicity in stochastic systems is concerned with a conceptual choice in giving meaning to quantitative probabilities. It can be argued that it is meaningless to assign a probability to a single event, and that any decision regarding a single event must resort to intuition or morals. For mathematical guidance, the event has to be embedded within other similar events. Fermat and Pascal (P. Fermat & B. Pascal 1654, personal communication between themselves) chose to embed within parallel universes, but alternatively—and often more meaningfully—we can embed within time. The concept of a decision regarding a single isolated event, whether probabilistic or not, seems dubious: how do we interpret the premise of isolation? Surely, the event is part of a history. Does the individual making the decision die immediately after the event? In general, the consequences of the decision will unfold over time.

The origins of ergodic theory lie in the mechanics of gases [15]. One is interested in large-scale effects of the molecular dynamics, i.e. in the thermodynamic variables. For instance, the macroscopic pressure of a gas is a rate per area of molecular momentum transfer to a container wall, averaged over an area that is large compared with the typical distance between molecules and over a time that is long compared with the typical interval between molecular impacts in the area.

As the number of particles is large and collisions are possible, however, it is practically not possible to explicitly solve the microscopic equations of motion. Full information about the state **x** (positions and momenta of all molecules) is not available, and the time average, for instance of momentum transfer to a container wall, cannot be computed directly. Boltzmann [16] and Maxwell [17] independently replaced the physically required time average by the average over an ensemble of appropriately weighted states **x**, making use of Huygens' expectation value. The weight of the different states **x** in the ensemble was postulated and subsequently justified empirically by comparing predictions with observations.

The key rationale behind this dramatic step is that the systems considered are in equilibrium: the macroscopic variables of interest do not change in time, and microscopic fluctuations obey detailed balance (e.g. van Kampen [18]). Under these strict conditions, time has little tangible effect, and we may get away with disregarding it completely. Nonetheless, both Boltzmann and Maxwell were concerned that for mathematical convenience they were using the *a priori* irrelevant ensemble average.

Specifically, when Boltzmann [16] suggested to treat a gas as a collection of many systems, namely subvolumes that can be thought of as a probabilistic ensemble, he warned that using this ‘trick’ means ‘to assume that between […] the various […] systems *no interaction ever occurs*’ (quoted in [9, p. 14]). The requirement of absolutely no interaction between a collection of systems is equivalent, in practical terms, to the non-existence of all these systems from each other's perspectives—if systems A and B cannot ever interact in any way, then to system A, for all practical purposes, system B does not exist and vice versa. Another way of putting this is that systems A and B are parallel universes.

Assuming the validity of this procedure is known as the ergodic hypothesis. It is permissible under strict conditions of stationarity (e.g. Grimmet & Stirzaker [19], ch. 9.5). These conditions were understood long after the St Petersburg paradox had been introduced [20–23].

Much of the literature on ergodic systems is concerned with deterministic dynamics, but the basic question whether time averages may be replaced by ensemble averages is equally applicable to stochastic systems, such as Langevin equations or lotteries. The essence of ergodicity is the question whether the system when observed for a sufficiently long time *t* samples all states in its sample space in such a way that the relative frequencies *f*(**x**,*t*) d**x** with which they are observed approach a unique (independent of initial conditions) probability, *P*(**x**) d**x**,
5.1
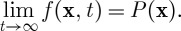


If this distribution does not exist or is not unique, then the time average, 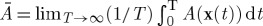, of an observable *A* cannot be computed as an ensemble average in Huygens' sense, 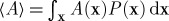. The generic variable *A* may depend on time only through its state dependence, or it may have explicit time dependence. If *P*(**x**) is not unique, then the time average of *A* generally depends on initial conditions. If *P*(**x**) does not exist, then there may still be a unique time average. A unique ensemble average may also still exist—although we cannot find *P*(**x**) from equation (5.1), we may be able to determine 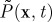, the proportion of systems in an ensemble that are in state **x** at time *t*, and compute the ensemble average as 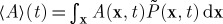. In special cases, the time dependencies of *A*(**x**,*t*) and 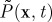 can be such that 〈*A*〉(*t*) does not actually depend on time. However, there is no guarantee in these cases that the time average and ensemble average will be identical.

Growth factors in the St Petersburg lottery are such a special case. In §6, it will be shown that the ensemble-average winnings (*a priori* irrelevant) from the lottery diverge, whereas the time-average winnings do not. Mathematically, the result is identical to the result obtained by Bernoulli (although see §7*a*). Conceptually, however, the arbitrary utility (arbitrary in the sense that it depends on personal characteristics) is replaced by an argument based on the physical reality of the passing of time and the fact that no communication or transfer of resources is possible between the parallel universes introduced by Fermat.

### (a) The economic context

To repeat, the quantity in equation (3.1) is accurately interpreted as follows: imagine a world of parallel universes defined such that every chance event splits our current universe into an ensemble containing member-universes for every possible outcome of the chance event. We further require that the proportion of members of the ensemble corresponding to a particular outcome is the probability of that outcome. In this case, if we give the same weight to every member-universe, then equation (3.1) is the ensemble average over all possible future states of the universe (i.e. states after the game).

Of course, we are not *a priori* interested in such an average because we cannot realize the average payout over all possible states of the universe. Following the arguments of Boltzmann and Maxwell, this quantity is meaningful only in two cases.

— The physical situation could be close to an ensemble of non-interacting systems which eventually share their resources. This would be the case if many participants took part in independent rounds of the lottery, with an agreement to share their payouts, which would be a problem in portfolio construction, and different from Bernoulli's set-up.^2^
— The ensemble average could reflect the time-average performance of a single participant in the lottery. Whereas time averages in statistical mechanics are often difficult to compute (hence the ergodic hypothesis), the simplicity of the St Petersburg lottery makes it easy to compute them and see how they differ from ensemble averages.

Thus, neither case applies to the St Petersburg lottery, and the ensemble average is irrelevant to the decision whether to buy a ticket.

In general, to realize an average over the ensemble, ensemble members must exchange resources, but this is often impossible, so we must be extremely careful when interpreting ensemble averages of the type in equation (3.1).

## 6. Resolution using non-ergodicity

The resolution of the St Petersburg paradox presented in this section builds on the following alternative conceptualization:

— *Rejection of parallel universes*. To the individual who decides whether to purchase a ticket in the lottery, it is irrelevant how he may fare in a parallel universe. Huygens' (or Fermat's) ensemble average is thus not immediately relevant to the problem.
— *Acceptance of continuation of time*. The individual regularly encounters situations similar to the St Petersburg lottery. What matters to his financial well-being is whether he makes decisions under uncertain conditions in such a way as to accumulate wealth *over time*.

Similarly, in statistical mechanics Boltzmann and Maxwell were interested in momentum accumulated *over time*. Because they considered equilibrium systems, where time is largely irrelevant, they hypothesized that time averages could be replaced by ensemble averages. However, a person's wealth is not usually in equilibrium, nor even stationary: on the time scales of interest, it generally grows or shrinks instead of fluctuating about a long-time average value. Therefore, the ergodic hypothesis does not apply [24]. Consequently, there is no reason to believe that the expected (ensemble-average) gain from the lottery coincides with the time-average gain. That they are indeed different will be shown in this section by explicitly calculating both.

The accumulation of wealth over time is well characterized by an exponential growth rate. To compute this, we consider the factor *r*_*i*_ by which a player's wealth changes in one round of the lottery,^3^
6.1
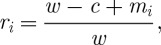
where, as in equation (4.1), $*w* is the player's wealth before the round of the lottery, $*c* is the cost of a lottery ticket and $*m*_*i*_ is the payout from that round of the lottery. To convert this factor into an exponential growth rate *g* (so that 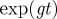 is the factor by which wealth changes in *t* rounds of the lottery), we take the logarithm, 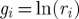.^4^

### (a) Ensemble average

**Theorem 6.1**

*The ensemble-average exponential growth rate in the St Petersburg lottery is* 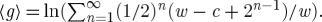

**Proof.**

First, we consider the ensemble-average growth factor, and begin by averaging over a finite sample of *N* players, playing the lottery in parallel universes, i.e. in general players will experience different sequences of coin tosses,
6.2
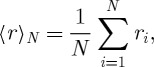
which defines the finite-sample average 〈·〉_*N*_. We change the summation in equation (6.2) to run over the geometrically distributed number of coin tosses in one round, *n*,
6.3
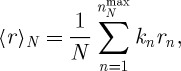
where *k*_*n*_ is the frequency with which a given *n*, i.e. the first tails-event on the *n*th toss, occurs in the sample of *N* parallel universes, and 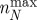 is the highest *n* observed in the sample. Letting *N* grow, *k*_*n*_/*N* approaches the probability of *n*, and we obtain a simple number, the ensemble-average growth factor 〈*r*〉, rather than a stochastic variable 〈*r*〉_*N*_
6.4
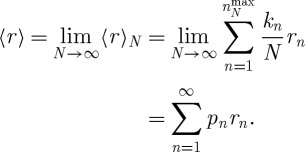
The logarithm of 〈*r*〉 expresses this as the ensemble-average exponential growth rate. Using equation (6.1) and writing the probabilities explicitly, we obtain
6.5
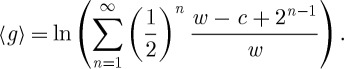

As the ensemble-average payout from one round in the lottery diverges, equation (3.1), so does this corresponding ensemble-average exponential growth rate equation (6.5).

### (b) Time average

**Theorem 6.2**

*The time-average exponential growth rate in the St Petersburg lottery is* 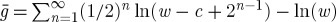
*.*

**Proof.**

The time average is computed in close analogy to the way the ensemble average was computed. After a finite number *T* of rounds of the game, a player's wealth reaches^5^
6.6
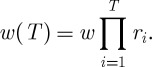
The *T*th root of the total fractional change,
6.7
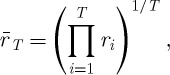
which defines the finite-time average 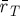, is the factor by which wealth has grown on average in one round of the lottery over the time span *T*. We change the product to run over *n*,
6.8
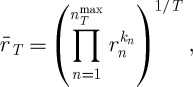
where *k*_*n*_ is the frequency with which a given *n* occurred in the sequence of *T* rounds, and 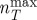 is the highest *n* observed in the sequence. Letting *T* grow, *k*_*n*_/*T* approaches the probability of *n*, and we obtain a simple number, the time-average growth factor 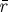, rather than a stochastic variable 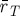

6.9
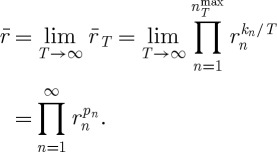

The logarithm of 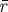 expresses this as the time-average exponential growth rate. Using equation (6.1) and writing the probabilities explicitly, we obtain
6.10
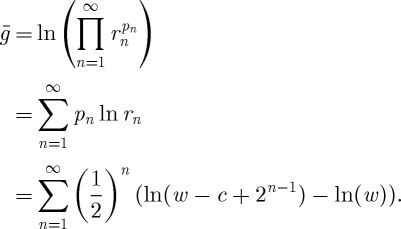

Equation (6.10)_3_ is identical to the right-hand side of equation (4.1).

**Figure 1.**
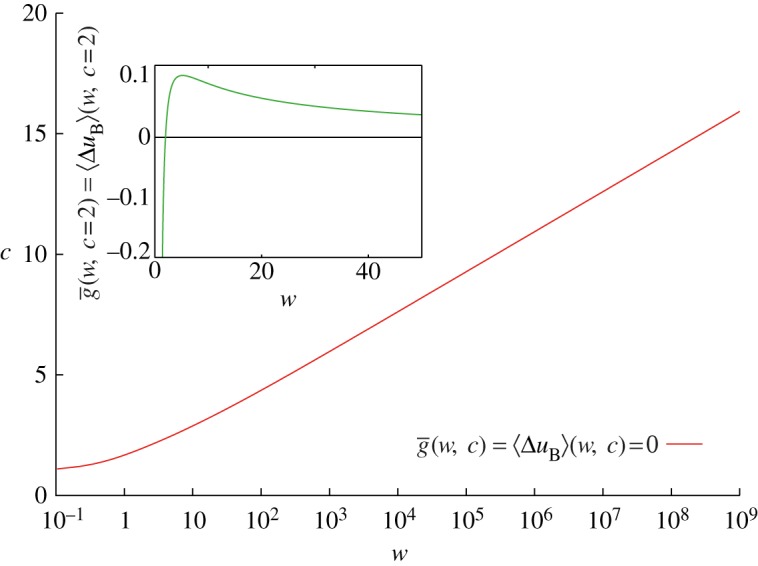
Equation (6.10) (or equation (4.1)) defines a relationship between *w* and *c*, where 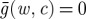, i.e. the player breaks even over time (or his ensemble-average logarithmic utility change is zero) if he pays $*c* given his wealth $*w*. Inset: time-average exponential growth rate (or ensemble-average logarithmic utility change), 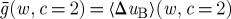, for the St Petersburg lottery as a function of wealth, $*w*, with a ticket price of $*c*=$2. If the player risks bankruptcy by purchasing a ticket, then 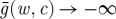. To the infinitely wealthy player, a gain or loss is irrelevant and 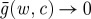. (Online version in colour.)

Again the quantity can be positive or negative, but, instead of the ensemble average of the change in utility, we have calculated the time-average exponential growth rate of a player's wealth without any assumptions about risk preferences and personal characteristics. If the player can expect his wealth to grow over time, and he has no other constraints, then he should play the game; if he expects to lose money over time, then he should not play. The loci of the transition between growth and decay, where 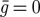, define a line in the *c* versus *w* plane, which is shown in figure 1. Equation (6.10) depends on the player's wealth—keeping *c*>1 fixed, 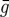 initially increases with *w*; see inset of figure 1 for the example of *c*=2. This is because the wealthy player keeps most of his money safe, and a loss does not seriously affect his future ability to invest. For the very wealthy player, neither a win nor a loss is significant, and the time-average exponential growth rate asymptotes to zero as 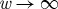. At the other extreme, a player whose wealth *w*≤*c*−1 risks bankruptcy, which in a sense means the end to his economic life, and 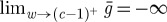.

So equation (6.10) can also be considered a criterion for how much risk a person should take. The cost of the ticket, *c*, is the exposure to the lottery. For fixed (positive) *w* ‘buying’, a ticket is always advisable if *c*=0; see §7*a*. As *c* increases, 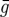 will eventually become negative as the exposure, or risk, becomes too large. The time resolution discourages entering into any gamble where bankruptcy, i.e. zero or negative wealth after the game, occurs with non-zero probability. In these cases, individual terms in the sum in equation (6.10) are undefined.

Equation (6.10) may seem an unnatural criterion for the following reason: the lottery is played only once with wealth *w*. By the next round, wealth has changed by *m*_*i*_−*c*, and the situation has to be re-evaluated. So in reality, although we may buy tickets at the same price repeatedly, a combination of different 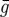 (resulting from different ‘initial’ wealths *w*) will be realized. However, over a sufficiently long time, we must assume that we will face equivalent decisions again, and thus play equivalent lotteries again. Let us consider the result of playing many rounds in different lotteries, 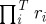. Because of commutativity, we can rearrange the factors in the product so that the first *T*′ factors correspond to the rounds in which we face equivalent lotteries (for instance, we have the same wealth, and the tickets are offered at the same price), and the remaining *T*−*T*′ factors refer to different situations,
6.11
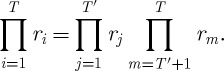
Whatever 
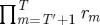
 may be, the steps in equation (6.9) apply to the first product, and the sign of the quantity in equation (6.10), which determines whether the first product is greater or smaller than 1, is a good criterion for deciding whether to buy a ticket.

It is instructive to calculate the time-average exponential growth rate in another way. Equation (6.10)_2_ looks like an ensemble-average exponential growth rate obtained by computing exponential growth rates for individual systems and then averaging those over the ensemble,
6.12
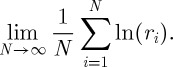


The reason why this quantity is not the ensemble average but the time average is subtle. There is no limit 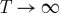, so how can this be a time average?

Averages extract deterministic parameters from stochastic processes. The ensemble average does this by considering an infinity of parallel universes, and the time average does it by considering infinitely many time steps. But to find the time average, it is not necessary for time itself to approach infinity. Instead, the unit of time can be rescaled. It is only necessary that all possible scenarios occur exactly with the appropriate frequencies during the sampling time. As long as the effect of time—of events happening sequentially—is accounted for, this will lead to the time average.

We have used one round of the lottery as one time unit. Thus, the return from one time unit will be one of the possible returns *r*_*n*_. If we observe only one time unit, then the best estimate for the time-average return would be the return that happened to be realized in that time step. An approximate estimate for the time-average exponential growth rate is thus 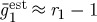.^6^

To improve this estimate, we pick *q* returns *r*_*j*_ at random and in agreement with the probabilities *p*_*n*_, and let each return act for 1/*q* time units.^7^ The total time that passes during the experiment is kept fixed but we separate it into *q* subintervals of time. The result will be
6.13
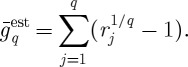
The proportion of subintervals during which return *r*_*n*_ is realized will approach *p*_*n*_ as 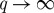. In this limit, we can therefore replace the sum over time steps by a sum over *n* as follows:
6.14
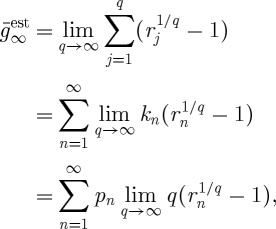
where *k*_*n*_ once again is the frequency with which a given *n* occurs, now in the sample of *q* subintervals. Using the definition of the logarithm, 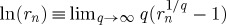 yields
6.15
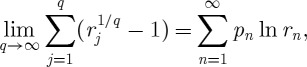
meaning that the time-average exponential growth rate, derived by splitting a time unit into infinitely many subintervals and playing through all possible scenarios in these subintervals, can be written as the expectation value of the logarithm of returns. A limit which is equivalent to the apparently missing limit 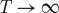 is implied by the logarithm and evaluated before the explicit limit in equation (6.12). Thus, equation (6.12) is an ensemble average of a time average, which is nothing but a time average.

## 7. Relation to Bernoulli's resolution

Equation (6.10) is mathematically equivalent to Bernoulli's use of logarithmic utility. Bernoulli argued behaviourally that, instead of the expectation value of monetary gain, the expectation value of the gain in a loosely constrained function (the utility) of wealth should be considered. One of the allowed functions is the logarithm, which has the special property of encoding the multiplicative nature common to gambling and investing in a linear additive object, the expectation value
7.1
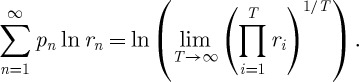
Inadvertently, by postulating logarithmic utility (left-hand side of equation (7.1)), Bernoulli replaced the ensemble-average winnings with the time-average exponential growth rate in a multiplicative non-ergodic stochastic process (right-hand side of equation (7.1)).

Bernoulli did not make the time argument, as is evident from his acceptance of Cramer's square-root utility, which does not have the property of equation (7.1): 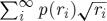 cannot be written as a similar product. This is problematic because the arbitrariness of utility can be abused to justify reckless behaviour, and it ignores the fundamental physical limits, given by time irreversibility, to what can be considered reasonable. But because Bernoulli's early work postulated the logarithm, many consequences of equation (6.10) have already been discussed in the literature under the heading of ‘logarithmic utility’.

A different heading for these results is the ‘Kelly criterion’ [25–27]. In contrast to ensemble-average exponential growth rates, which often diverge (for instance with leverage), time-average exponential growth rates can be optimized [24,28]. Kelly [25] used this fact to optimize wager sizes in a hypothetical horse race using private information. While he refrained from using utilities because he deemed them ‘too general to shed any light on the specific problems' he considered [25, p. 918], he did not point out the fundamental difference in perspective his treatment implies: in essence, arbitrary utility functions are replaced by the physical truth that time cannot be reversed. My aim here is to emphasize this difference in perspective. It is crucial that logarithmic utility from this point of view is not a utility at all. Rather, the logarithm accounts for the multiplicative nature of the process: the ensemble average of the logarithm of growth factors equals the logarithm of the time average of growth factors.

Comparing equation (6.10) and equation (4.1), it is tempting to say that the time average justifies logarithmic utility. I advise against this interpretation because it conflates physical concepts of time with behavioural concepts of usefulness. Any utility function other than the logarithm leads to recommendations that do not agree with the time perspective.

### (a) Menger's objection to unbounded utility

Bernoulli [1] did not actually write down equation (4.1), although it is often assumed that that was his intention. Instead of using the criterion equation (4.1), he argued in two steps ‘how large a stake an individual should be willing to venture’ [25], pp. 26–27.

— The expected gain in utility is calculated without explicitly taking the ticket price into account
7.2
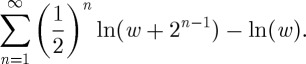
— This is followed by the statement that ‘the stake more than which persons […] should not venture’ is that ticket price, $*c*, which satisfies
7.3
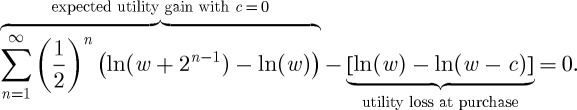

This is not the condition that defines the line in the main panel of figure 1 as it does not imply that the expected net change in utility, equation (4.1), is zero. In this sense, equation (4.1) is an inaccurate, although generally accepted and sensible, representation of Bernoulli's work. The difference between equation (4.1) and equation (7.3) and the conflicting interpretations of these equations are consequences of the aforementioned arbitrariness of the utility framework.

Menger [2] claimed that using logarithmic utility as Bernoulli did, equation (7.2) and equation (7.3), does not resolve modified versions of the paradox, where payouts $*f*(*n*) as a function of waiting time *n* increase faster than according to Bernoulli's original *f*_*B*_(*n*)≡2^*n*−1^. Specifically, Menger considered 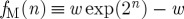. Note that this function is curiously defined in terms of the initial wealth. His first step closely mirrors Bernoulli's first step, but then he jumps to an untenable conclusion:

— Menger [2] pointed out that, by replacing 2^*n*−1^ in equation (7.2) by *f*_M_(*n*), the expected gain in logarithmic utility at zero ticket price diverges.
— He argued that the paradox remains unresolved because ‘it is clear that also in the modified St. Petersburg game no normal person will risk a large amount or even his fortune as a wager’ ([2, p. 468], my translation), and generalized to conclude that this formally prohibits the use of any unbounded utility function.

The meaning of the second statement is unclear. A player who pays ‘his fortune’ for a ticket in Menger's lottery and then experiences a heads-event on the first coin toss, i.e. the worst possible outcome, will still gain since the worst-case payout, 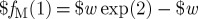, is more than the initial wealth, $*w*. For a person to risk losing anything at all, the ticket price has to be $*c*>$*f*_M_(1), far greater than the person's wealth. For a person to risk losing his entire wealth, the ticket price has to be greater still, 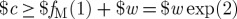. But at such prices equation (7.3) is undefined.

Perhaps Menger meant that Bernoulli's condition, equation (7.3), cannot be satisfied, and the price one should be willing to pay is infinite. In that case, the second part of Menger's argument implicitly assumes that the positive divergence in the first part cannot be offset by anything else. But as the ticket price approaches the player's wealth, *c*→*w*, the utility loss at purchase in equation (7.3) represents another divergence, implying that this argument is inconclusive. It will now be shown to be invalid.

Menger's objection to Bernoulli could hold in the following sense: the left-hand side of equation (7.3), using Menger's payout function, diverges positively if *c*<*w* and is undefined otherwise. But it is never zero (or negative)—when does it warn against the gamble? To understand what the undefined regime signifies, one has to study the process of divergence and compare the two infinities as they unfold.

For any finite 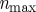, a finite value *c*<*w* does exist, which renders the corresponding partial sum zero,
7.4
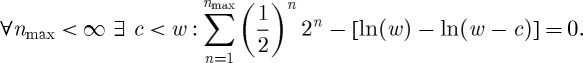
To ensure positivity up to exactly *c*=*w*, where the expression becomes undefined, events of zero probability have to be taken into account. For any non-zero lower bound on probability, Bernoulli's condition can be satisfied. In this sense, in the literal original Bernoulli set-up, values of *c*≥*w*, where equation (7.3) is undefined, correspond to the recommendation not to buy a ticket, and the paradox is resolved.

Menger's conclusion is incorrect. Bernoulli's logarithmic utility recommends to purchase tickets as long as they cost less than the player's wealth, implying a significant minimum gain—a course many a ‘normal person’ may wish to pursue. The criterion could be criticized for the opposite reason: it rejects prices that guarantee a win, even in the worst case.

The time resolution produces the criterion in theorem 6.2, which is equivalent to equation (4.1) and not to Bernoulli's literal original criterion equation (7.3). Consequently, it yields a different recommendation, which may at first appear surprising but turns out also to correspond to reasonable behaviour given the assumptions on which it is based: it recommends to purchase a ticket at any price that cannot lead to bankruptcy. The player could be left with an arbitrarily small positive wealth after one round. The recommendation may be followed by a ‘normal person’ because of the assumption that equivalent lotteries can be played in sequence as often as desired. Under these conditions, irrespective of how close a player gets to bankruptcy, losses will be recovered over time. Of course, if these conditions are violated, the time resolution does not apply. This last statement is another warning against the naive use of mathematics, whose truth is always restricted to the context of axioms or assumptions. Applicability reflects the degree to which assumptions are representative of real conditions in a given situation. While ensemble averages are meaningless in the absence of samples (here parallel rounds), time averages are meaningless in the absence of time (here sequences of equivalent rounds).

## 8. Discussion

Excessive risk is to be avoided primarily because we cannot go back in time. Behavioural aspects and personal circumstances are relevant on a different level—they can change and do not immediately follow from the laws of physics.

The perspective described here has consequences far beyond the St Petersburg paradox, including investment decisions [24,28] as well as macro-economic processes. For example, it is sensible for a nation striving for growth to encourage risks that lead to occasional bankruptcies of companies and individuals. How large a risk is macroeconomically sensible? What are the moral implications? Does gross domestic product—a linear sum, similar to a sample average—measure what one should be interested in? While the St Petersburg lottery is an extreme case, equation (6.10) and figure 1 carry a more general message: if net losses are possible, the negative time-average exponential growth rate for small wealth, *w*, turns positive as *w* increases, implying higher exponential growth rates for larger entities. In a collection of such entities, inequality has a tendency to increase, letting large entities dominate and monopolies arise. This can cause markets to cease functioning, as competition is compromised or corporations become ‘too big to fail’. There is anecdotal evidence that assets in stock markets have become more correlated in recent decades, and effective diversification (which mimics ensembles) harder to achieve. This would make the time perspective even more important, and the consequences of ignoring it more dramatic.

Utility functions are externally provided to represent risk preferences, but are unable by construction to recommend appropriate levels of risk. The framework is self-referential in that it can only translate a given utility function into actions that are optimal with respect to that same utility function. This can have unwanted consequences. For example, leverage or credit represents a level of risk that needs to be optimized, but current incentive structures in the financial industry can encourage exceeding the optimal risk. Adam Smith [29], cited in Foley [30], warned that excessive lending—in his case based on bills of exchange for goods in transit—can lead to a collapse of the credit system, followed by bankruptcies and unemployment; on the other hand, insufficient lending can lead to economic stagnation, two stages that often follow one another in a boom–bust cycle. To avoid both, good criteria for appropriate levels of risk are needed, which the utility framework cannot deliver. The time arguments presented here provide an objective null-hypothesis concept of optimality and can be used to optimize leverage under a given set of conditions [24]. In the present case, optimality based on such considerations is a good approximation to practically optimal behaviour. This is evident from Bernoulli's work, whose justification of the almost identical result was practical validity.

The proposed conceptual re-orientation may help reconcile economic theory with empirical regularities, such as risk aversion, known from behavioural economics.

It is easy to construct examples where less risk should be taken than recommended by the criterion in equation (6.10). For example, some fraction of $*w* may already be earmarked for other vital use. It is very difficult, however, to think of an example where greater risk is beneficial. For this reason, the time perspective is a useful tool for finding upper limits on risk to be implemented in regulation, for instance as margin requirements, minimum capital requirements or maximum loan-to-value ratios. Of course, such regulation must take into account further arguments, but its least complex form can be based on the time perspective.

The epistemological situation is typical of the process of concept formation [31]. As the conceptual context changed, in this case from moral to predictive, the original definition of the term ‘expectation’ began to lead to paradoxical conclusions. From today's conceptually later perspective, it appears that N. Bernoulli made a hidden assumption, namely the assumption, explicitly stated by Huygens [11], that ‘it is the same thing’ to receive five shillings as it is to have an equal chance of receiving either three or seven shillings. Lakatos [31] points out that it can be hard to imagine in retrospect that an eminent mathematician made a hidden assumption, which is often perceived as an error. He writes on p. 46, ‘while they [the hidden assumptions] were in your *subconscious*, they were listed as *trivially true*—the … [paradox] however made them somersault into your conscious list as *trivially false*’. Similarly, it seems trivially true at first that the expected gain from playing the lottery should be the criterion for participating—taking time into account makes this assumption somersault into being trivially false.

Thus, the St Petersburg paradox relies for its existence on the assumption that the expected gain (or growth factor or exponential growth rate) is the relevant quantity for an individual deciding whether to take part in the lottery. This assumption can be shown to be implausible by carefully analysing the physical meaning of the ensemble average. A quantity that is more directly relevant to the financial well-being of an individual is the growth of an investment over time. Utility, which can obscure risks, is not necessary to evaluate the situation and resolve the paradox. It is the actual wealth, in $, of a player, not the utility, that grows with 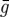 (equation (6.10)). It is manifestly not true that the commonly used ensemble-average performance of the lottery equals the time-average performance. In this sense, the system is not ergodic, and statements based on anything other than measures of the actual time-average performance must be interpreted carefully.

## Footnotes

1 See Devlin [5] for a detailed historical account.

2 This situation is equivalent to a single person buying tickets for many parallel rounds of the lottery. In the limit of an infinitely rich person and a finite ticket price it can be shown that it is advisable to invest all funds in such independent lotteries.

3 One ‘round’ of the lottery is used here to mean one sequence of coin tosses until a tails-event occurs. Throughout, an index *i* refers to such rounds, whereas *n* indicates waiting times—the number of times a coin is tossed in a given round.

4 The logarithm is taken to facilitate comparison with Bernoulli's analysis. As long as it acts on the average, as opposed to being averaged over, it does not change the convergence properties.

5 A possible objection to this statement is discussed below, starting on the next page.

6 The approximation  is permissible here because we will consider infinitesimal time steps such that  will be exact.

7 The subscript *j* is used here instead of *i* because it refers not to one round but to subintervals of one round.
